# Even the Thinnest Salami Contains Some Meat

**DOI:** 10.5811/westjem.2018.4.38408

**Published:** 2018-04-12

**Authors:** Paul Walsh

**Affiliations:** Sutter Medical Center Sacramento, Department of Emergency Medicine, Division of Pediatric Emergency Medicine, Sacramento, California

In this issue of the journal, Condella *et al* describes apparently vast differences between emergency departments (ED) in the use of albuterol for infants with bronchiolitis who were sufficiently ill as to require admission to the hospital.^1^ This study is a secondary analysis of a subset of patients admitted to the hospital or pediatric intensive care unit in the Multicenter Airway Research Collaboration (http://www.emnet-usa.org). The data is relatively old and pre-dates the current American Academy of Pediatrics (AAP) guideline to not even attempt albuterol use in these patients.^2^

At first blush the difference in albuterol use is striking. In some centers as few as 23% of children destined for admission received albuterol; in others, 84% did. Given that the AAP guidelines at the time advised a therapeutic trial of albuterol for bronchiolitis, the obvious question is why was albuterol not tried in everyone?

It is tempting to point to the almost-religious zeal with which some groups oppose albuterol use in these patients. The dogma appears odd given that random controlled trial evidence in fact favors a trial of albuterol in these patients.^3^ Meta analyses were crafted that excluded studies which found decreased admissions with albuterol.^4^–^6^ Null analyses with a power as low as 18% have been mischaracterized as evidence to not use albuterol.^6^–^7^ When even these select studies showed that albuterol decreased respiratory distress in infants with bronchiolitis, “relief of respiratory distress” was dismissed as “not patient centered.”^2^ Perhaps this is the culture to which Condella *et al* refers when trying to explain its findings.

Other reasons may be the natural history of bronchiolitis and the heterogeneity in its diagnosis. The natural history of bronchiolitis is broadly this: inoculation (day #0) with a swift rise in prostanoid production (possibly triggering apnea^8^), followed by cough and runny nose starting on day #3. This is followed on days #3 to #5 by gradual-onset wheezing in the lung bases, which progresses throughout the lungs and from day #5 is accompanied by the development of crackles in the lower lung bases. The disease peaks in severity about day #7 to #9 post-inoculation by which stage crackles heard first become predominant throughout all lung fields before gradually resolving from days #10 to #14. Each of these stages of bronchiolitis invites different treatments, and even different diagnoses. In the upper respiratory tract infection-phase stage, albuterol seems unlikely to help. Later phases may attract diagnostic terms such as viral-induced wheeze, wheezy bronchitis, reactive airway disease and even asthma, rather than bronchiolitis. When a child has wheezing albuterol is more likely to be prescribed, and by the time the child has predominantly crackles the doctor may believe that there is no point trying albuterol.

The inclusion criteria of the parent study do not help. Although Condella *et al* refers to the description of bronchiolitis in the 2004 AAP guidelines, the inclusion criteria of the parent study required that the patient have a “physician diagnosis of bronchiolitis.” Some physicians may interpret (in an unfortunately circular logic) a response to albuterol as evidence against bronchiolitis. So, at least some of the difference between EDs’ use of albuterol may reflect heterogeneity in diagnosis.

The actual recruitment over a three- to four-year period from some of these sites was very low (range 28 to 139 patients). To a community pediatric emergency physician 28 bronchiolitics sounds more like a single busy shift rather than three to four years of recruitment. With such low numbers from each site there is concern that neither the study patients nor the diagnostic decision-making are representative of infants who attend for bronchiolitis. The authors provide no data to reassure us on this point.

Another reason for the apparent starkness of the differences is the way in which the authors present their data. Condella *et al* uses bar charts of percentages, which do not account for the total number of patients recruited at each site. Here we re-draw Condella *et al’s* Figure as a funnel plot to show how such data can be better presented.^9^ Over-dispersion observed in funnel plots is commonly seen when unmeasured covariates are not taken into account.^10^

In our clinical experience many children have in addition to a mixture of crackles and wheezes any number of other ill-defined adventitial noises. Unsurprisingly, interrater agreement for auscultatory findings in bronchiolitis is low.^11^ These adventitial sounds often improve with albuterol. The accompanying improvement in respiratory distress is often incomplete; even if wheezing resolves, the increased work of breathing often persists. Still, the improvement in respiratory distress is sometimes sufficient to enable safe discharge.

The authors fitted a logistic regression model to explore the relative role of different independent variables that predict the use of albuterol. As might be expected wheezing was associated with more, and duration of illness longer than 7 days with less, albuterol use). Unfortunately, the authors did not take this (analytically straightforward) step further and estimate the probability of a range of typical patients at varying stages of bronchiolitis receiving albuterol at each ED. Plotting these results by ED may have shown the apparent differences to diminish given similar patients. Other quirks in the analysis, such as the reversal of some associations in bivariate and multivariable analysis, remain unaddressed.

Sometimes, as section editors for *WestJEM* we receive manuscripts that have been presented elsewhere prior to reaching our desks. These manuscripts may well have been improved by the input of other reviewers prior to reaching us. However, sometimes we see unwelcome influences and in this manuscript the authors felt the need to state they agree with the AAP guidelines in their abstract’s conclusion despite their study not assessing the effect of albuterol. Too often the evidence shows what the most powerful person in the room says it shows. Worse, authors feel the need to genuflect accordingly or remain unpublished. We reviewers and editors are not blameless.

So, what does Condella *et al* offer the practicing emergency physician?

1. An insight into the likely heterogeneity in the diagnosis of bronchiolitis in academic EDs.
2. Evidence of a determination in some academic EDs to not use albuterol in bronchiolitis even when AAP guidelines recommended a therapeutic trial. Presumably convinced of the correctness of their own position (evidence not withstanding) this group felt themselves in no way bound by the AAP guidelines of the time. Community emergency physicians should feel similarly empowered today.
3. (Yet more) evidence of some corners of academia pushing the thinnest of salami papers with the least effort that they can get away with while genuflecting towards power and tenure committees rather than advancing knowledge.

So why publish? First, 1 and 2 are informative for emergency physicians who find the current AAP recommendation to not attempt a therapeutic trial of albuterol at odds with their own experience that albuterol sometimes helps. Second, Condella *et al* demonstrates to future trialists that standardized diagnostic criteria or analysis adjustment based on clinical descriptors of the illness could improve future bronchiolitis research.

## Figures and Tables

**Figure:**
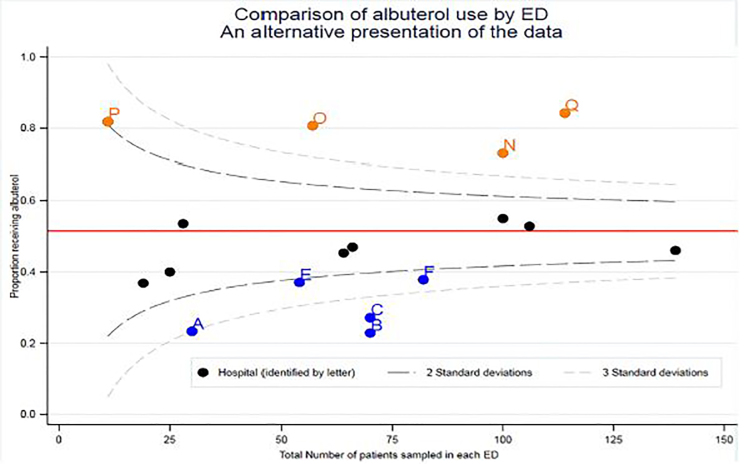
This funnel plot showing outliers by two and three standard deviations addresses the difficulty of comparing performance when the denominator varies between individual sites. It does not address limitations of the underlying, data-generating mechanism. Data here has been redrawn from Figure 1 in Condella et al. The over-dispersion observed here suggests important unmeasured or unadjusted covariates. *ED,* emergency department.
